# Historic review: a short history of neuropediatrics in Germany between 1850 and 1950

**DOI:** 10.1186/s42466-020-00072-2

**Published:** 2020-09-01

**Authors:** Hans Michael Strassburg

**Affiliations:** 1 Gerbrunn, Germany; 2 Former Universitäts-Kinderklinik Würzburg, Josef Schneiderstr. 2, 97080 Würzburg, Germany

**Keywords:** Child neurology, Neuropediatrics, 1850 till 1950, Ludwig Mauthner, Georg Peritz, Philipp Schwartz, NS-era

## Abstract

Since the middle of the nineteenth century, there has been increasing knowledge about the diagnosis and therapy of diseases of the central and peripheral nervous system and muscle in children. The leading causes were cerebral palsy, epilepsy, inflammatory and degenerative diseases, and the innate reduction in intelligence. Because of the often lack of healing options, many pediatricians had little interest in treating these children and left their care to the pedagogues and psychologists. Pioneers of child neurology in the German-speaking countries were Ludwig Mauthner, Franz von Rinecker, Julius Zappert, and Georg Peritz. Especially with the beginning of the National Socialist terror regime, rigorous treatment methods were used, the care facilities for disabled children were closed, and these were either actively murdered or interned under inhumane conditions. In this time, some specialists in neuropediatrics had an ambivalent position between the care for their patients and the selection for their elimination. After 1950, new findings in diagnostics and therapy, especially from Anglo-American countries, played a significant role in the rise of neuropediatrics in Germany.

## Background

Neuropediatrics or child neurology includes the diagnosis and treatment of congenital and acquired diseases of the central and peripheral nervous system and muscles in children and adolescents. Historical aspects of individual neuropediatric clinical symptoms were published in many places, especially about epilepsy and cerebral palsy. Especially after 1950, unexpected advances in diagnostics and therapy were achieved through the development of entirely new techniques, particularly electrophysiology, imaging, and molecular genetics. Nevertheless, it is worth looking back at the history of the field to get to know its roots better.

## Introduction

In the medical literature of the late nineteenth century, the diagnoses cretinism, syphilis, and rickets were primarily used for congenital disabilities, which according to modern ideas, are no longer comprehensible and which hide many very different clinical symptoms. In public and, not least, by representatives of the churches, children with developmental disorders and disabilities were viewed as a punishment from God or as a result of “original sin” well into the twentieth century. They were repeatedly associated with an allegedly sinful lifestyle on the part of the parents [1, 2]..

From today’s perspective, other explanations for congenital and early acquired “disabilities” were severe epilepsy, metabolic disorders (e.g., PKU), poliomyelitis, tuberculosis, injuries to the central nervous system, bacterial meningitis, brain tumors, brain malformations, especially hydrocephalus, and various causes of deafness and blindness, and much more. Children with “cretinism” not only have congenital hypothyroidism but also a variety of other causes of the developmental disorder, e.g., have had chromosomal abnormalities and different syndromes [1–5].

### First exact descriptions of neurological diseases in children

**Fig. 1:**
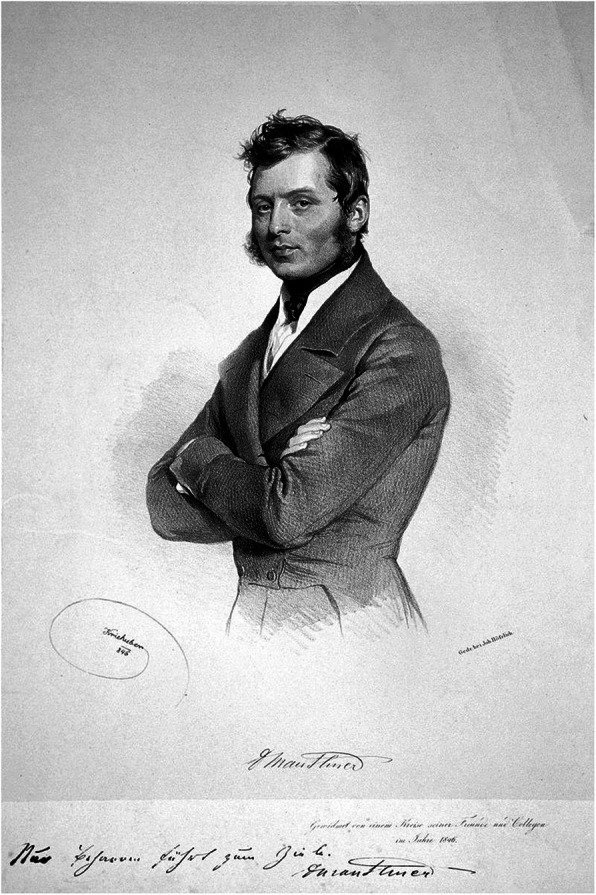
Ludwig Wilhelm Mauthner von Mauthstein. Lithograph by Josef Kriehuber (wikipedia 4.5.2020)

Ludwig Wilhelm Mauthner (later von Mauthstein, 1806–1858), the founder of the first children’s hospital in the German-speaking area in Vienna, published the first neuropediatric monograph “The diseases of the brain and spinal cord in children” in 1844 with detailed descriptions of his autoptic findings. Mostly the children died of infections like congenital syphilis and especially disseminated tuberculosis, but the main reason for death, according to Mauthner, was poverty [6] (Fig. 1).

**Fig. 2:**
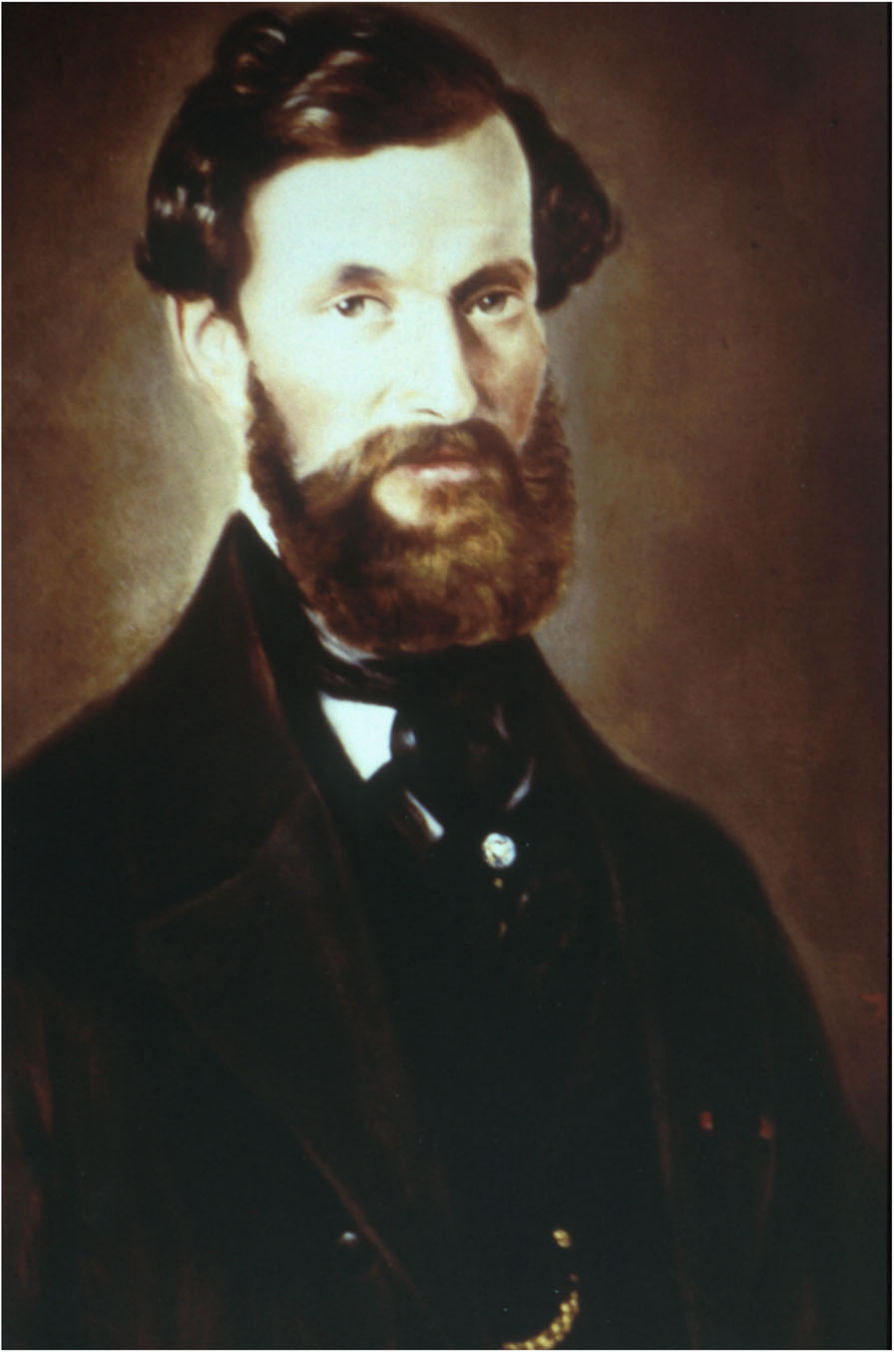
Franz von Rinecker (photograph of the Children’s University Hospital Wuerzburg)

The Würzburg professor of pediatrics Franz von Rinecker (1811–1883) published many casuistic lectures on neuropediatric patients, e.g., about cerebrospinal meningitis, encephalitis, pachymeningitis, “essential polio” and the “madness of the children”. In 1850 he founded the first stable children’s university hospital in Germany, but at the same time also taught his students in internal medicine, pharmacology, dermatology, and psychiatry. Particularly noteworthy is his report published in 1859 on “Cases of General Muscular Pseudohypertrophy in Boys”, the first German description of progressive muscular dystrophy. However, the English doctor Edward Meryon (1807–1880) had already described this disease in 1852. But progressive muscular dystrophy is named after Guillaume Duchenne de Boulogne (1806–1875), who published extensively about 13 patients in 1868 (Fig. 2).

The internist Carl Gerhardt (1833–1902), an academic student of von Rinecker in Würzburg and later chief of the internal medicine hospital in Berlin, was the editor for the first extensive manual of pediatrics with several articles on neurological diseases by various authors [1, 7].

The Frankfurt psychiatrist Heinrich Hoffmann (1809–1894) described in his worldwide successful children’s book “Der Struwwelpeter” published in 1844 children with various, mainly child and adolescent psychiatric and neuropediatric disorders such as ADHD (“Zappelphilipp”), the socio-oppositional behavioral disorder (“The Evil Friederich”), the excessive thumb sucking (“Konrad der Daumenlutscher”) and the absence epilepsy (“Hans Guck in the air”) without claiming a “scientific contribution”. However, he pointed out that children are not “small adults”, but that they have to be treated as independent personalities with special needs and diseases [8].

In 1867, a “care facility for epileptic boys” was inaugurated in Bethel near Bielefeld. A few years later, in 1872, Pastor Friedrich von Bodelschwingh (1831–1910) took over the leadership and developed it into one of the most important institutions of the Inner Mission. Because of the “hopelessness of the medical treatment”, especially in the patients with epilepsy, he saw his main task in the “pastoral-pedagogical leadership” [1, 2].

### Otto Heubner and Sigmund Freud

The first director of a children’s university hospital in Germany, Otto Heubner (1843–1926), described in a famous textbook of pediatrics published in 1906 with a total of 1227 pages on a mere 144 pages neurological diseases highlighting the poor differentiation of the various causes of congenital disabilities. He repeatedly emphasized the great difficulties of her treatment but also mentioned incredible treatment successes with dried and powdered thyroid tissue in some cases.

He explained about hydrocephalus (p. 98): “Such children go early, within the first few years of their lives, but … complete insanity and animal vegetation are more common.”

On “simple severe idiocy”, he wrote (p. 129): “In this stupid state, which is far below the normal animal, the child can spend years, decades until a catastrophe from frequent seizures or an infectious disease puts an end to life” [9].

In the last third of the nineteenth century, significant progress was made, particularly in the detection of neurological diseases in adults. This understanding increasingly found its way into pediatrics. Sigmund Freud (1856–1939) worked for 15 years at the first public children’s hospital in Vienna with cerebral palsy in children and published several articles, most extensively in 1897. He is the founder of the international classification of cerebral palsy that is still valid today and differentiated between prenatal and “intra- and postpartum” causes and particularly pointed out the importance of damage during pregnancy. In his opinion there was no causal connection between cerebral palsy and epilepsy but he thought on neurosurgical interventions for epilepsy. He repeatedly expressed frustration with his work, including in a letter from 1890: “The completely uninteresting work on child-like paralyzes took up all my time” [1, 10].

### Epidemiology of developmental disorders and disabilities in children around 1900

Since the middle of the nineteenth century, representatives of the Prussian army pointed out the inadequate health of the young men who were moved into military service. At the same time, many institutions for the medical treatment of sick children were founded in many German cities by women from the aristocracy and the wealthy citizens.

The general practitioner and orthopedist Konrad Biesalski (1868–1930) fundamentally improved the living conditions of many people with physical disabilities. He founded and directed, among others, the Oskar-Helene-Heim in Berlin, the largest private orthopedic institution for children and adolescents, and he carried out a first “cripple count” in Germany. He promoted rehabilitation and special education for them, not least to train young people or get them back into a paid profession. In 1906 there were, according to his information, 600,000 “crippled children” in Germany - in 1910, there were around 400 “auxiliary schools” for about 25,000 “mentally underdeveloped children”.

His motto was: “Not a single foot should be treated, but the whole person”. However, he rigorously refused to support mentally impaired people: “Epileptics and idiots are worthless human material” [2].

### The “utility concept”

At least since the end of the nineteenth century in many countries, there have been considerations of how a person can be assessed in terms of its “usefulness”, e.g., in England by T. Malthus and F. Galton. As a result of the evolutionary theory of Charles Darwin, Ernst Haeckel (1834–1919), Alfred Ploetz (1860–1940), etc. advocated this theory in Germany. As well in Scandinavia and the USA, the reproduction of “inferior, sick” people should be prevented by sterilizing them, and thus the development of a higher quality “breed” made possible. These ideas culminated after the catastrophe of the First World War with millions of young men killed and crippled in 1920 in the well-known writing by the lawyer Karl Binding (1841–1920) and the psychiatrist Alfred Hoche (1865–1943) “The release of the annihilation of unworthy life. Its measure and its form” [2, 11].

### Specialization

**Fig. 3:**
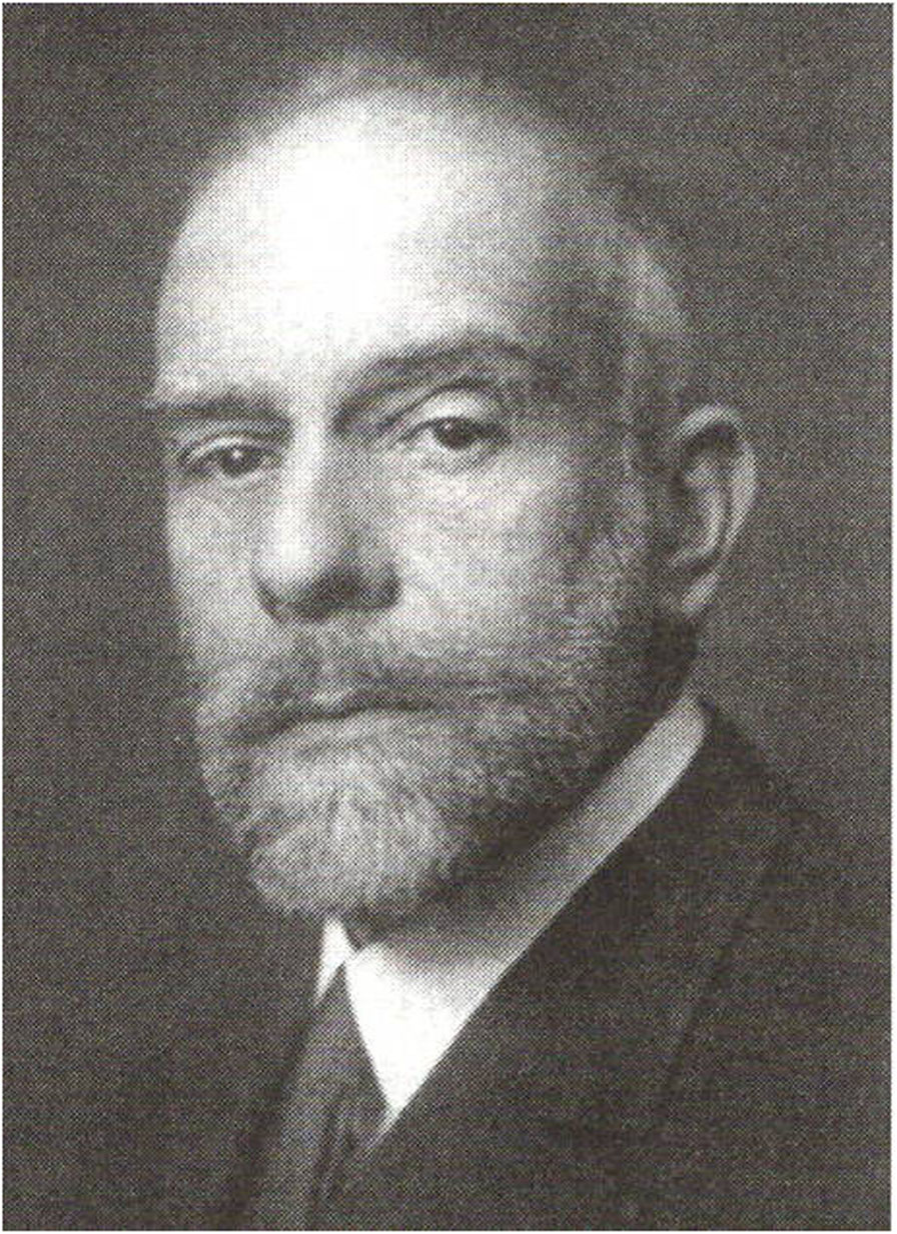
Julius Zappert (from 21)

At the beginning of the twentieth century, the Austrian pediatrician Julius Zappert (1867–1942) published an extensive contribution about the diseases of the nervous system in childhood in the Handbook of Childhood Diseases, edited in 1906 by Meinhard von Pfaundler and Arthur Schlossmann [12] (Fig. 3).

Above all, Georg Peritz (1870–1935), primarily specialized in endocrinology, published two detailed monographs on the knowledge at that time about diseases of the nervous system in children. In the 1912 edition he used the following classification:

- Infantile cerebral palsy
- The familial, hereditary and congenital diseases of the central nervous system
- The inflammatory diseases
- The neuroses
- The diseases of the central nervous system as a result of functional disorders of the glands and internal secretion
- The congenital psychoses [13]

**Fig. 4:**
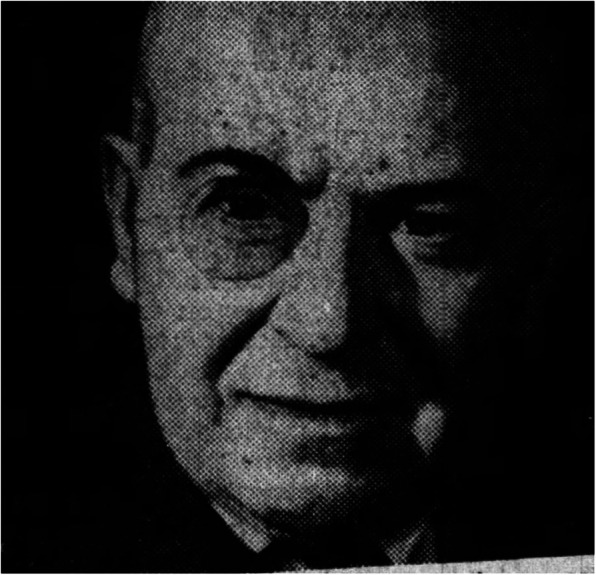
Georg Peritz (from 25)

**Fig. 5:**
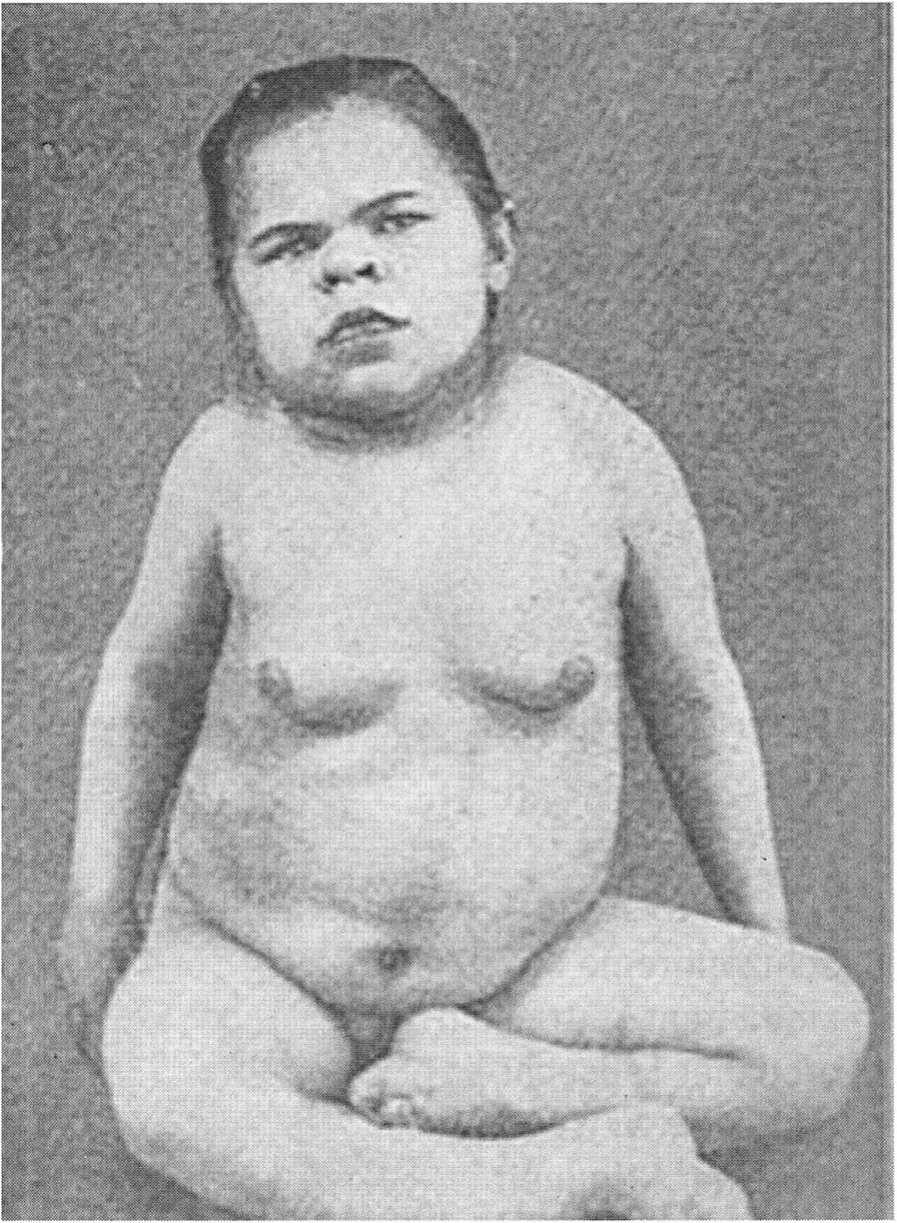
Patient with severe myxedema by congenital hypothyreoidism (from 16)

Peritz, like his teacher, the founder of adult neurology in Germany, Hermann Oppenheim (1857–1919), primarily dealt with pathological-anatomical findings and the influence of the then known hormones on the CNS. For example, epilepsy in children often expresses a “spasmophilic constitution” with disturbances of the calcium-metabolism. Neither the first nor the completely revised second edition of 1932, is mentioned in later reviews, even after the second world war, and he is not registered as a lecturer for childhood diseases in Berlin. Was it because of his Jewish background? [13, 14] (Figs. 4 and 5).

**Fig. 6:**
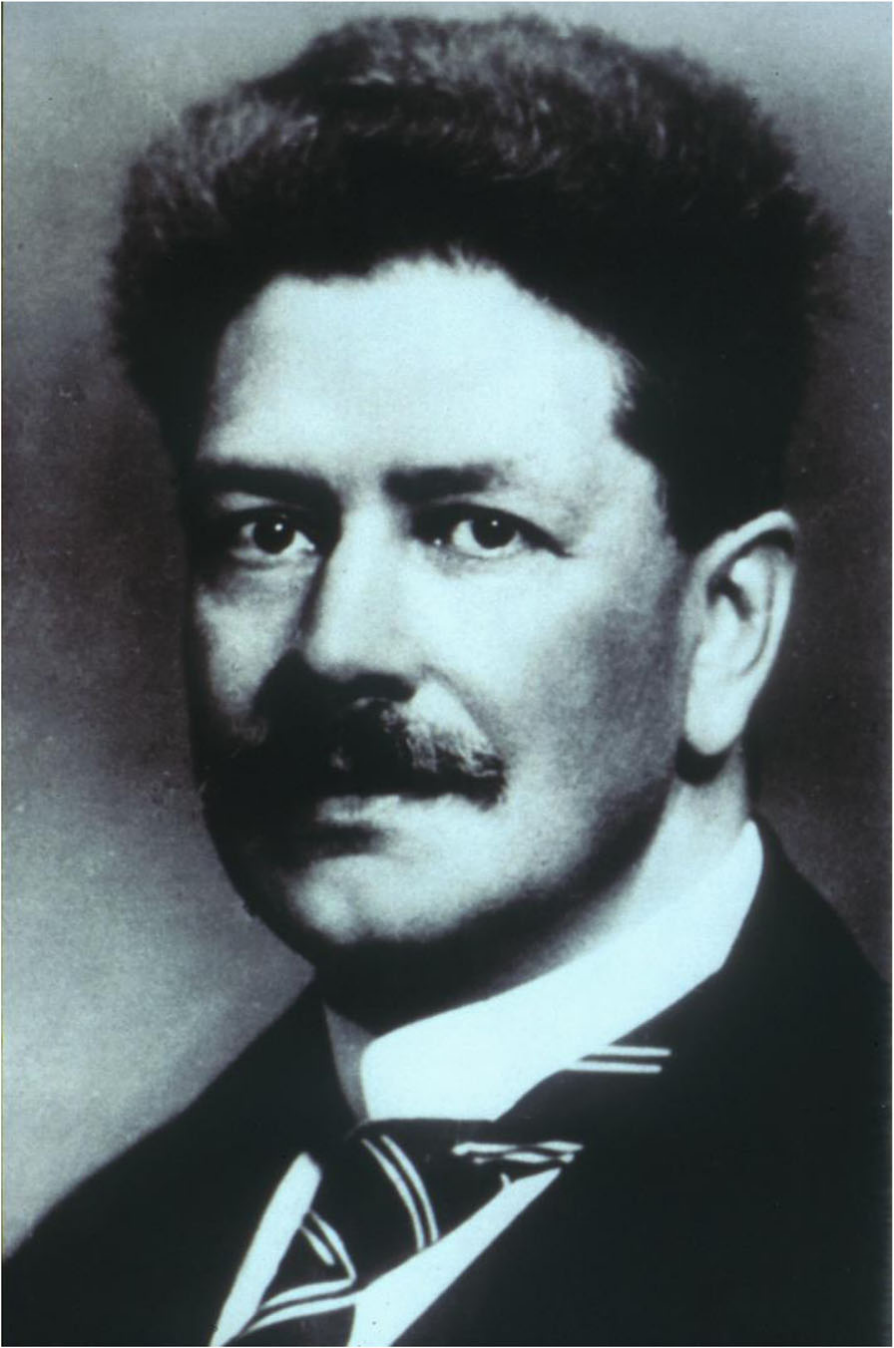
Yussuf Ibrahim (photograph of the Children’s University Hospital Wuerzburg)

Yussuf Ibrahim (1877–1953), a long-time director of the Children’s University Hospital Jena, described children with peculiar neurological symptoms, e.g., tics and psychogenic paralysis. Because of his contribution in the 1931 Handbook of Pediatrics, also edited by M. von Pfaundler and A. Schlossmann, on “Organic Diseases of the Child’s Nervous System”, he was described by some of his students, e.g., Hans Rudolf Wiedemann, the director of the Children’s University Hospital in Kiel, as the “founder of German neuropediatrics”. The proof that he transferred children with severe mental disabilities to the Stadtroda “Kinderfachabteilung”, knowing about their fate, impaired his high reputation [11] (Fig. 6).

In summary, in the first decades of the twentieth century, the medical treatment options for severe neurological diseases and disabilities were minimal, and the doctors were very reluctant to deal with these patients. Antibiotics or effective anti-epileptics were not available, only in exceptional cases, e.g., in hypothyroidism or acute rickets, causal treatments have improved significantly. Many of these children died prematurely from infections, in particular bacterial pneumonia and meningitis, nutritional disorders, heart, and respiratory failure and prolonged cerebral attacks, but also from senseless and dangerous treatment measures such as serial puncture of hydrocephalus, overdoses of opiates and after 1912 even from Barbituric acid derivatives [1, 15].

### Examples for good care

Children with congenital and acquired movement disorders were treated in the “cripple institutions” founded in 1830 with various, sometimes drastic “aids” and the first operations provided, so by the founder of the German orthopedics Jakob von Heine (1800–1879) [2, 3].

The former officer Detleff Neumann-Neurode (1879–1945), in cooperation with pediatricians and orthopedic surgeons, introduced therapeutic gymnastics to infants and young children in Berlin before the First World War and pointed out the importance of children’s sport for the prevention and treatment of malpositions and functional disorders, e.g., as a result of rickets. He also supported his treatment method for “slightly mentally disabled” children [1, 16, 17].

The St. Josef House in Gemünden on Main, which was founded in 1882 by the physician Johann Michael Herberich (1854–1930), was an example of the exemplary care and support for “mentally disabled” children and young people. In his 1910 published monograph, “Methodology of the weak-minded. For use in teachers’ seminars, auxiliary schools, and institutions as well as in private lessons”, he describes his practical experience in the institution, which had an international reputation. In 1941 the facility was closed rapidly by the National Socialists, and the children were either transferred directly to murder-places such as Pirna-Sonnenstein or, under inhumane conditions, to psychiatric state hospitals [2, 11].

### The “Nuremberg Laws”

In 1933, at the beginning of the Nazi dictatorship, about half of the pediatricians in the German-speaking world were Jewish, especially in social pediatric and university institutions. They were immediately released from civil service, were usually only able to work in private practices for a short time, had to emigrate, were locked up in concentration camps, transported to extermination camps or voluntarily put an end to their lives.

On July 15, 1933, the “Law for the Prevention of the Offspring with Hereditary Disease “, also known as the “Nuremberg” Hereditary Health Act”, was published in the Reichsgesetzblatt. It says:

§ 1: If you are inherited, you can be sterilized by surgery if, based on the experience of medical science, it is highly likely that your offspring will suffer severe physical or mental inheritance damage.

For this law, a person who is suffering from one of the following diseases is a hereditary disease:

- congenital idiocy
- schizophrenia
- circular insanity
- hereditary epilepsy
- hereditary St. Vitus dance
- hereditary blindness
- hereditary deafness
- severe hereditary physical malformation.
- Furthermore, those who suffer from severe alcoholism can be rendered sterile [2, 11].

### Reichs committee and T4 action

By decree of the Ministry of the Interior of August 18, 1939, midwives, obstetricians, nurses, and other doctors involved were obliged to report all children with “congenital malformations and mental underdevelopment” to the responsible health authority up to the age of 3. The sheets were sent to the “Reichsausschuss zur wissenschaftlichen Erfassung von erb- und anlagebedingten schweren Leiden” (Reichs Committee for the Scientific Assessment of Hereditary Serious Sufferings“ (RA). There the pediatricians Werner Catel from Leipzig (1891-1981), Hans Heinze from Brandenburg-Görde (1895-1983), and Ernst Wentzler from Berlin (1891-1973) assessed with “enthusiasm and conviction”.

Independently of this, children and adolescents cared for in medical and nursing homes were also included in the “Action T4”, the systematic murder of mentally ill and disabled people, which was carried out independently of the RA procedure. In a personal letter dated September 1, 1939, Adolf Hitler had ordered SS doctor Karl Brandt and Reichsleiter of the NSDAP Philipp Bouhler to organize the killing of severely disabled people, which he cynically described as “death by grace”. The children who were positively reported by the RA and T4 experts were transferred to so-called children’s departments under the pretext of getting into “facilities for the best care and the possibilities of modern therapies”. From there, many of them were transported under great secrecy in killing facilities such as Grafeneck, Hadamar, Bernburg, Brandenburg-Görde, and Pirna-Sonnenstein, or murdered directly by administering high-dose barbiturates or morphine, sometimes also by intracardiac injections of phenol [2, 11].

An example of how to deal with children who suffered from malformations is the contribution by H.C. Hempel from the Children’s University Hospital Leipzig, whose director was the Reich Committee expert W. Catel, on the “Spina bifida cystica - clinic, therapy, and population policy significance”. In it, he advocated surgery only for small, deep-seated celes without significant neurological deficits. Children with pronounced celes and hydrocephalus should be left to their fate and should not be operated [18].

### Child neurology in Germany between 1933 and 1945

In 1942 the “Textbook of Pediatrics” by Philipp Bamberger et al. dealt with the diseases of the nervous system on 71 of 821 pages, of which the malformations on 3 pages, the epilepsy on 4 pages and the intelligence deficits on 1 page [19].

The professor at the Pediatric University Hospital in Gießen, Walter Keller (1894–1967 - after 1949 director of the Freiburg University Hospital), wrote in this textbook (pages 45–63): “Since the reporting of these sufferings not only serves to recognize the diseased genetic material in our national body but in many cases can answer important questions for the later decision of the Hereditary Health Court, the need for timely recognition and diagnosis of relevant conditions is evident.” One was very uncertain with the so-called “cerebral palsy” or “Little’s disease”, which in part was explained on “exogenous damage before and during birth, partly due to hereditary-constitutional dispositions” [19]..

In the same book, Rudolf Degwitz (Hamburg, 1889–1973) wrote an additional, 8-page chapter “on the education and treatment of neuropathic and psychopathic children”. He described “... primitive, affect-emphasized, irrational, subcortical, drives to gain pleasure and avoid feelings of discomfort, which are only described as psychopathic and neurotic in people whose behavior, like that of schoolchildren, adolescents and adults, is age-appropriate their objective insight into reality. In the case of toddlers, the higher intellectual classes are developing, however, the possibility of an objective recognition of reality still lacks due to its purely affective, ego- and anthropomorphic position to the world, so these reactions are to be regarded as physiological”. - “... such reactions gradually disappear in an educationally favorable milieu” (pp.719–727) [19].

“Behavioral problems” such as sleep disorders, loss of appetite, recurrent vomiting, umbilical colic, finger sucking, nail-biting, nasal drilling, wetting, caking and poor concentration were among others explained with vegetative hyperexcitability and mainly seen as a result of educational errors. Also, with “neuropathic persons” there would occasionally be frequent “absences”, shortly diminished consciousness (up to 100 and more per day), which, in contrast to the epileptic absences, would not lead to a change of character without leaving any traces. These would spontaneously disappear and were called “pyknoleptic seizures”. They are “bromine and luminal resistant”. Also, neurotics would produce large seizures at appropriate moments that were almost entirely similar to the epileptic grand mal (pp. 719–727) [19].

### The situation of pediatrics after 1945

After the end of World War II and the atrocities of the medically and politically responsible persons only gradually becoming known, the improvements of general living conditions, especially overcoming famine, reducing infant mortality, and treating acute infectious diseases, e.g., tbc and poliomyelitis, were in the foreground of pediatric interests. There was no functioning public health system, many of the clinics were bombed out and without qualified personnel, and many pediatric practices were orphaned.

When filling the chairs and chief physician positions for pediatrics, only a few pediatricians were found who were not involved in the machinations of the National Socialists (including Bremen, Erlangen, Hamburg, Würzburg etc.). Personalities who were classified as “followers” despite their openly represented fascist ideas were often employed to regain leading positions, e.g. in Berlin, Freiburg, Göttingen and Münster. Quite blatantly, the former senior consultant of the “Reich Committee for the Scientific Assessment of Hereditary Illnesses” and Director of the Leipzig Children’s University Hospital Werner Catel was appointed director of the Children’s University Hospital in Kiel in 1954. There he was able to continue public the representation of his ideology of killing “life not worth living” [20, 21].

### Diseases and treatment options around 1950

**Fig. 7:**
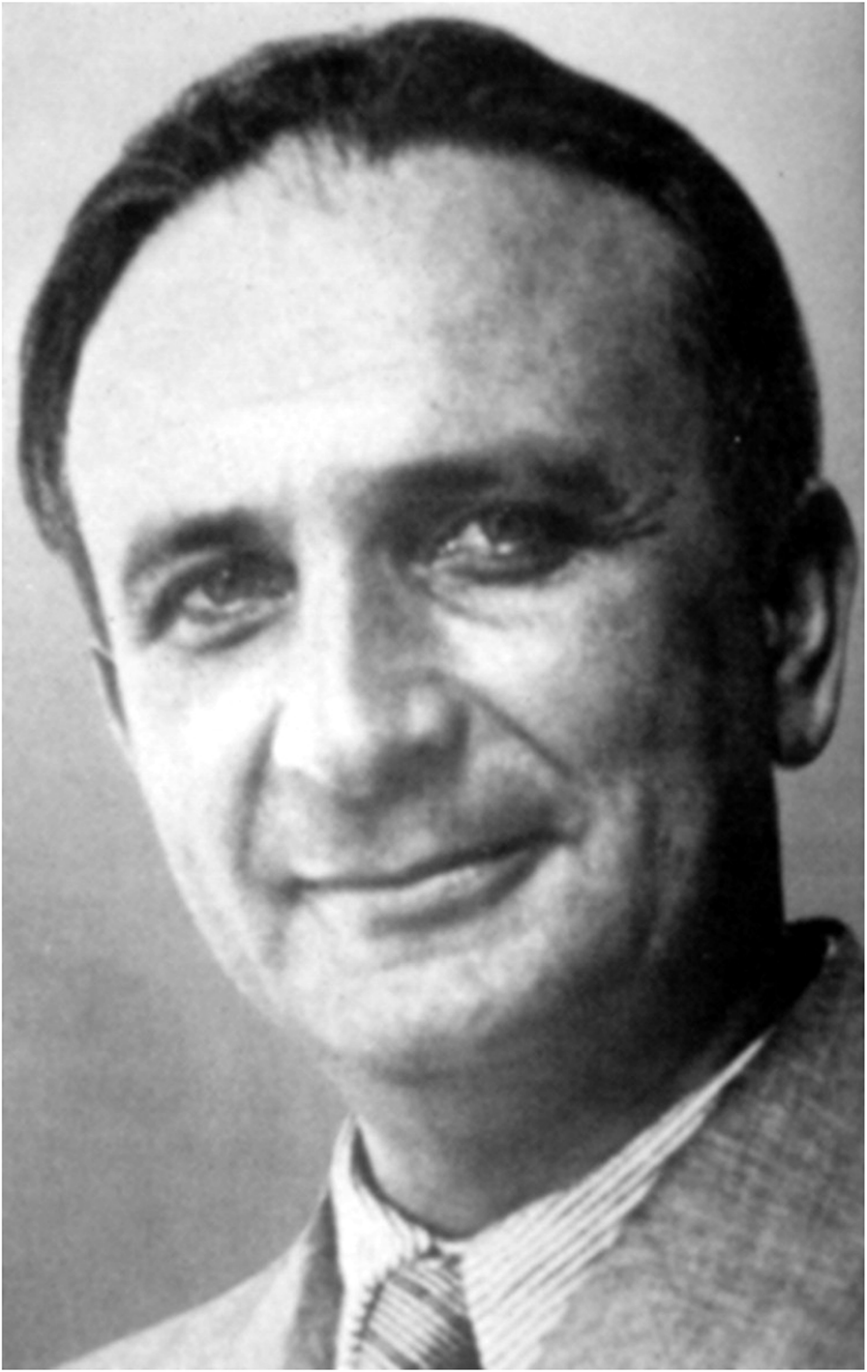
Philipp Schwartz (link.springer.com)

In 1924 and in a much more extensive edition in 1964, the neuropathologist Philipp Schwartz (1894–1977), who originally worked in Frankfurt and emigrated after 1933 via Switzerland to Ankara and later to the United States, described traumatic brain damage during childbirth as the most crucial explanation for a large number of severe and minor cerebral dysfunction. This means a counter position to the overemphasis on hereditary disorders [11, 22] (Fig. 7).

But there were also some other wrong turns: already the highly respected Rudolf Virchow (1821–1902) described the newborn as a “typical example of a spinal cord system”. British Nobel laureate Lord C.S. Sherrington (1857–1952) and the Utrecht physiologist Rudolf Magnus (1873–1927) founded the theory of positional reactions with their investigations on differently decerebrated animals. The pediatrician Albrecht Peiper (1889–1968) and the neurologist Georges Schaltenbrand (1897–1979), both were involved in the Nazi-ideology, continued this concept. It was the basis for the outdated teaching on the caudo-cranial development of the human nervous system and the importance of reflexology in infants and young children for the early diagnosis of movement disorders [1, 23].

In 1918, Walter Edward Dandy (1886–1946) described pneumencephalography for the first time. The German internist Adolf Bingel (1879–1953) improved this technique, and it was used extensively between 1930 and 1960 for diagnosing brain-tumors or -malformations, but mostly moderate knowledge gain in children with developmental and behavioral problems [24].

Objective diagnostic methods for testing brain functions, e.g., the EEG, were only gradually introduced in German children’s hospitals after 1950. Here the publications of Richard Jung (1911–1986) and Dietrich Pache (1911–1978) in addition to the international literature of William G. Lennox (1884–1960) and Frederic A. Gibbs (1903–1992) were groundbreaking [1, 5].

Till the end of the second world war, there was little collaboration with clinical psychologists and pedagogues in children with conspicuous behavior, except some units for pediatric curative education, e.g. at the Children’s University Hospital Vienna, which was founded by Clemens von Pirquet (1874–1929) in the 1920s. Intelligence diagnostics was only gradually beginning to find its way into pediatrics; the experiences of the Vienna psychologist Charlotte Bühler (1893–1974) and their collaborators were not yet generally known [2, 4].

Medicines to treat common epilepsy were primarily bromine and phenobarbital (Luminal^R^) and increasingly hydantoin after 1950 [2, 11, 14].

In pediatric neurosurgery, there were only a few treatment options for brain tumors or hydrocephalus, mostly with side effects, especially since many excellent neurosurgeons with Jewish descent had to leave Germany after 1933 [25].

## Conclusion on the history of neuropediatrics in Germany in the first half of the twentieth century

Until 1933 there were many improvements for the situation of children with nerval diseases in the field of clinical diagnostics as well as in prevention and therapeutic care by improvements in curative education, the foundation of specialized children’s hospitals, and several social pediatric institutions, especially by Jewish pediatricians.

Between 1933 and 1945, only a few pediatricians opposed the utilitarian eradication craze of the National Socialist ideology in Germany [11, 20].

Already in 1897, the “Treatise on the nervous diseases of children” by Bernhard Sachs was translated into German and influenced the knowledge of neurological diseases in childhood. It was followed by the extensive standard work of Frank R. Ford (1892–1970) “Diseases of the Nervous System in Infancy, Childhood, and Adolescence”, which was published in four editions between 1937 and 1952 [26, 27]. Since 1950, international scientific pediatrics has made unprecedented advances in the prevention, diagnostics, and therapy of children with diseases of the nervous system which could be adopted in Germany more and more.

However, there are still major scientific and ethical problems today, e.g.

- in prenatal diagnosis and the resulting consequences
- with new treatment methods, e.g., fetal surgery, neonatology, intensive care and especially gene therapy
- when defining the limits of medical measures, e.g., for long-term ventilation of severely disabled patients
- in the inclusion of children with impairments in kindergarten and school, but especially in the society
- the costs incurred for new treatments and rehabilitation measures and
- in providing explanations for the relatives and their acceptance of the realities, e.g., with severe genetic abnormalities and congenital malformations.

## Data Availability

Not applicable.

## Abbreviations

ADHD: Attention deficit hyperactivity disease
EEG: Electroencephalography
NS: National Socialist
NSDAP: National Socialist German Workers Party
PKU: Phenylketonuria
RA: Reichsausschuss zur wissenschaftlichen Erfassung von erb- und anlagebedingten schweren Leiden
SS: Schutz Staffel (Protection Squad)
T4: Thiergartenstrasse 4
